# Control of mitochondrial dynamics and apoptotic pathways by peroxisomes

**DOI:** 10.3389/fcell.2022.938177

**Published:** 2022-09-09

**Authors:** Chenxing Jiang, Tomohiko Okazaki

**Affiliations:** ^1^ Graduate School of Pharmaceutical Sciences, The University of Tokyo, Tokyo, Japan; ^2^ Laboratory of Molecular Cell Biology, Institute for Genetic Medicine, Hokkaido University, Sapporo, Hokkaido, Japan

**Keywords:** peroxisomes, mitochondria, fission-fusion, apoptosis, organelle interaction, Zellweger syndrome, tethering

## Abstract

Peroxisomes are organelles containing different enzymes that catalyze various metabolic pathways such as β-oxidation of very long-chain fatty acids and synthesis of plasmalogens. Peroxisome biogenesis is controlled by a family of proteins called peroxins, which are required for peroxisomal membrane formation, matrix protein transport, and division. Mutations of peroxins cause metabolic disorders called peroxisomal biogenesis disorders, among which Zellweger syndrome (ZS) is the most severe. Although patients with ZS exhibit severe pathology in multiple organs such as the liver, kidney, brain, muscle, and bone, the pathogenesis remains largely unknown. Recent findings indicate that peroxisomes regulate intrinsic apoptotic pathways and upstream fission-fusion processes, disruption of which causes multiple organ dysfunctions reminiscent of ZS. In this review, we summarize recent findings about peroxisome-mediated regulation of mitochondrial morphology and its possible relationship with the pathogenesis of ZS.

## Introduction

Peroxisomes are single membrane-bound organelles found in almost all eukaryotic cells and have several essential metabolic roles in cell physiology. They contact other organelles such as mitochondria, the endoplasmic reticulum (ER), and lysosomes in various physiological and pathological contexts. In particular, peroxisome-mitochondria interactions play collaborative roles in fatty acid metabolism, redox homeostasis, and antiviral responses. Mitochondria are dynamic organelles whose morphology continuously changes. Cells activate an apoptotic program in response to stresses, such as DNA damage or signals during development, which is regulated by the mitochondrial apoptotic pathway. Mitochondria trigger apoptosis by releasing proteins located in the intermembrane space into the cytosol. However, it was unclear until recently whether peroxisomes are involved in these mitochondria-related phenomena. Based on the findings mainly in mammalian cells, this review discusses the newly discovered regulatory roles of peroxisomes in mitochondrial fission-fusion dynamics and intrinsic apoptotic pathways.

## Peroxisomes

### Basic functions of peroxisomes

Peroxisomes are organelles surrounded by a lipid monolayer and their diameter ranges from 0.1 to 1 μm^1^. They are responsible for many metabolic functions such as α- and β-oxidation of fatty acids, metabolism of reactive oxygen species (ROS) and reactive nitrogen species (RNS), and biosynthesis of ether-phospholipids and bile acids (Trompier et al., 2014; Wanders, 2014). To drive these metabolic functions, peroxisomes contain various types of enzymes such as catalases and peroxidases for ROS degradation and acyl-CoA oxidases, bifunctional proteins and thiolates for fatty acid catabolism (Fujiki, 2016; Fransen et al., 2017; Islinger et al., 2018; Fujiki et al., 2020). In addition, peroxisomes have non-metabolic roles such as antiviral defense and combat pathogens (Islinger et al., 2018).

### Peroxisomal proteins: Peroxins

Proteins involved in the biosynthesis and functions of peroxisomes are collectively called peroxins (the encoding genes are called *PEXs*). Their roles can be broadly classified into three categories: 1) peroxisomal membrane formation and membrane protein transport, 2) protein transport from the cytosol to the peroxisomal matrix, and 3) peroxisomal division and proliferation (Fujiki et al., 2020).

Basically, peroxisomal membrane proteins (PMPs) are transported through the cytosol to peroxisomes after being translated. In the class I pathway, cytosolic Pex19 binds to the newly synthesized PMPs and carries them to the peroxisomal membrane. In this pathway, Pex3, a membrane protein on peroxisomes, serves as the membrane-anchoring site for Pex19 and PMP complexes (Fujiki et al., 2020). In the class II pathway, nascent Pex3 is transported to the peroxisomal membrane (please refer to a recent review for more details about these Pex19-dependent pathways (Fujiki et al., 2020)). Pex3 is crucial for formation of peroxisomal membrane structures and therefore its genetic disruption results in failure of peroxisome formation (Muntau et al., 2000).

Although Pex19 was believed to explain the targeting of all PMPs, a recent study by Dahan et al. (2022) identified a Pex19-independent pathway. They found that specific PMPs are translated beside peroxisomes and disturbance of this process induces incomplete maturation of peroxisomes and concomitant impairment of cellular functions in yeast. Localized translation on peroxisomal membranes might ensure accurate and efficient membrane protein targeting because the authors found that mistargeting of peroxisomal mRNAs to different destinations such as the ER and mitochondria can disturb the functions of proteins encoded by the transcripts (Dahan et al., 2022). Although neither the amino acid sequences nor the protein structures that define this local translation process of PMPs have been elucidated, their work proposes how targeting specificity could be achieved by localized translation of specific transcripts proximal to peroxisomal membranes (Dahan et al., 2022).

Two specific peroxisome-targeting sequences play key roles in transport of peroxisomal matrix proteins: PTS1 (peroxisomal targeting signal 1) at their C-terminus and PTS2 at their N-terminus (Fujiki et al., 2020). Pex5, one of the peroxins, has been reported to be involved in the process of matrix protein transport. The shorter form of Pex5 called Pex5S binds to PTS1, whereas the longer form, Pex5L, acts as a receptor for PTS2 by making a cargo complex with Pex7 (Fujiki et al., 2020), thereby transporting synthesized peroxisomal matrix proteins from the cytoplasm to the peroxisomal matrix (Fujiki et al., 2020). Therefore, genetic disruption of Pex5 can lead to loss of peroxisomal matrix proteins (Baes et al., 1997).

The peroxisomal division process comprises three steps: elongation, constriction, and fission (Fujiki et al., 2020). In the elongation process, polyunsaturated docosahexaenoic acid promotes hyper-polymerization of Pex11β and leads to formation of Pex11β-enriched regions, which initiate peroxisomal elongation in one direction (Itoyama et al., 2012). Next, in the constriction and fission processes, Mff (mitochondrial fission factor) and Fis1 (mitochondrial fission protein 1), tail-anchored proteins which are also targeted to mitochondria, localize to the membrane-constricted areas of elongated peroxisomes, where Mff and Fis1 recruit Drp1 (dynamin-related protein 1) from the cytosol. A recent study indicates that Mff and Fis can act independently in this peroxisome division process (Schrader et al., 2022).

Since Drp1 requires a large amount of GTP as an energy source (Fujiki et al., 2020), mechanisms to supply and regulate this energy resource are important. In *Cyanidioschyzon merolae*, the nucleoside diphosphate kinase-like protein DYNAMO1 provides GTP pool (Imoto et al., 2018). In the first step of division, DYNAMO1 colocalizes with Drp1 and they form a ring structure called the peroxisome-dividing machinery (as peroxisomes share the machinery with mitochondria, the structure is also called mitochondria-dividing machinery) (Imoto et al., 2018). This machinery has a diameter of 50–600 nm and is composed of dynamin-based rings and skeletal filamentous rings (Imoto et al., 2013). DYNAMO1 converts cytosolic ATP into GTP locally at the peroxisomal-dividing machinery to produce GTP pool (Imoto et al., 2018). Upon the GTP generation, the ring-like structure generates a strong driving force that constricts and pinches peroxisomes (Imoto et al., 2018). After the fission process has finished, the ring structure composed of DYNAMO1 and Drp1 is immediately disassembled (Imoto et al., 2018).

### Peroxisome-related diseases

Reflecting the essential roles of peroxisomes in cellular metabolism, deficiency of peroxisomal function often causes severe diseases in mammals. Peroxisome-related diseases are classified into two groups: 1) peroxisome biogenesis disorders (PBDs), which are caused by mutations of *PEX* genes, and 2) single peroxisomal enzyme deficiencies (Trompier et al., 2014). Regarding PBDs, 14 *PEXs* have been identified as causative genes for peroxisomal dysregulation, and mutations of these genes are thought to cause various metabolic abnormalities (Fujiki et al., 2020). Thirteen out of the identified PBDs risk genes have been identified to cause Zellweger spectrum disorder (ZSD) (Fujiki et al., 2020). ZSD is further classified into the most severe type of Zellweger syndrome (ZS), the less severe neonatal adrenoleukodystrophy (NALD), and milder infantile Refsum disease (IRD) (Trompier et al., 2014) (More precise information on the disease clarification is summarized in Fujiki et al., 2020). Especially, ZS is characterized by multiple organ dysfunctions. Patients exhibit defects such as in the brain, liver, kidney and skeletons, and eventually die within a few months after birth (Goldfischer et al., 1973). Peroxisomes thus play an essential role in maintaining the functions of various tissues. In addition, alterations of mitochondrial ultrastructures have been observed in cells derived from patients with ZS, suggesting that there is a link between the pathologies of ZS and disruption of mitochondrial morphology (Baumgart et al., 2001).

## Regulation of mitochondrial morphology

### Mitochondrial fission-fusion

Mitochondria are dynamic organelles that continuously divide and fuse in a highly regulated manner. These morphological changes are vital for mitochondrial inheritance, mitochondrial quality control, and maintenance of mitochondrial functions (Wang et al., 2020). For example, it is well known that mitochondria changes their morphologies dynamically along with the cell cycle (Mishra and Chan, 2014). While enhanced fusion is associated with the G1 to S phase transition, mitochondrial fission is activated during mitosis (Mishra and Chan, 2014). Mitochondrial dynamics are also dynamically affected by various factors such as diet (Putti et al., 2015), redox homeostasis (Willems et al., 2015; Brillo et al., 2021) and infection (Tiku et al., 2020).

Several GTPases are involved in regulation of mitochondrial morphology (Detmer and Chan, 2007), and interestingly, some of them including Drp1, Fis1 or Mff are shared by both mitochondria and peroxisomes (Fujiki et al., 2020). During the mitochondrial fission process in mammalian cells, cytosolic Drp1 is recruited by its receptors, such as Mff, Mid49/51 (mitochondrial dynamics 49/51), and Fis1, to mitochondrial outer membranes (Giacomello et al., 2020). At the fission site, Drp1 forms a ring-shaped multimeric complex and utilizes energy generated by hydrolytic activity of GTP to contract the ring, causing mitochondrial cleavage (Giacomello et al., 2020).

Two GTPases, Mfn1 (mitofusin 1) and Opa1 (optic atrophy protein 1), which localize to the outer and inner membranes, respectively, play important roles during the fusion process. The fusion process is initiated by docking of two Mfn1 molecules in *trans*. Then, outer membranes of different mitochondria fuse and this is mediated by hydrolysis of GTP by Mfn1. At the same time, the inner membrane protein Opa1 binds to cardiolipin, a phospholipid present in the other mitochondrial inner membrane, in a heterotypic *trans* way to promote inner membrane fusion (Giacomello et al., 2020).

The physiological importance of mitochondrial dynamics has been widely discussed in recent years, and accumulating reports imply that mutated fission-fusion factors cause several diseases including neurodegenerative disorders (Van Laar and Berman, 2009; Giacomello et al., 2020). For example, neurons from individuals with Alzheimer’s disease exhibit broken cristae, near-total loss of inner structures, and a decreased number and increased volume of mitochondria (Zhu et al., 2013). In toxin-induced Parkinson’s disease models, donut-shaped mitochondria are observed in HeLa cells (Benard et al., 2007), increased fragmentation is observed in human fibroblasts (Mortiboys et al., 2008), and rapid fragmentation of mitochondria is observed in rat cortical neurons (Barsoum et al., 2006; Van Laar and Berman, 2009). In Huntington’s disease, mitochondrial dynamics are unbalanced toward fission in lymphoblasts and striatal precursors (Costa et al., 2010; Guedes-Dias et al., 2016). These reports suggest that the fission-fusion balance of mitochondria should be precisely maintained to keep cells healthy.

### Peroxisomes affect mitochondrial fission-fusion dynamics

Recent studies have revealed that organelles function not only in their own organizations but also by communicating with other organelles. The heterotypic organelle juxtapositions are called membrane contact sites (MCSs), where outer membranes of organelles do not fuse but are in close contact at a distance of 10–80 nm^31^. Thanks to technological advances in fluorescent proteins and microscopies, organelle contacts have been intensively studied in the last few decades. Identification and functional analysis of proteins forming MCSs have indicated that MCSs are involved in the transport of lipids, ions, and amino acids between organelles and the subcellular localization, growth, and division of organelles themselves (Scorrano et al., 2019; Schrader et al., 2020). Moreover, recent reports have illustrated that MCSs also have roles in regulating organelle morphology (Friedman et al., 2011; Korobova et al., 2013; Wong et al., 2018; Tanaka et al., 2019; Abrisch et al., 2020). For example, mitochondria-ER contact sites (MERCs) can regulate mitochondrial morphology, both by regulating fission (Friedman et al., 2011; Korobova et al., 2013; Abrisch et al., 2020) and fusion (Abrisch et al., 2020) processes of mitochondria. MCSs between lysosomes and mitochondria have also been reported to regulate the mitochondrial fission process (Wong et al., 2018).

**FIGURE 1:**
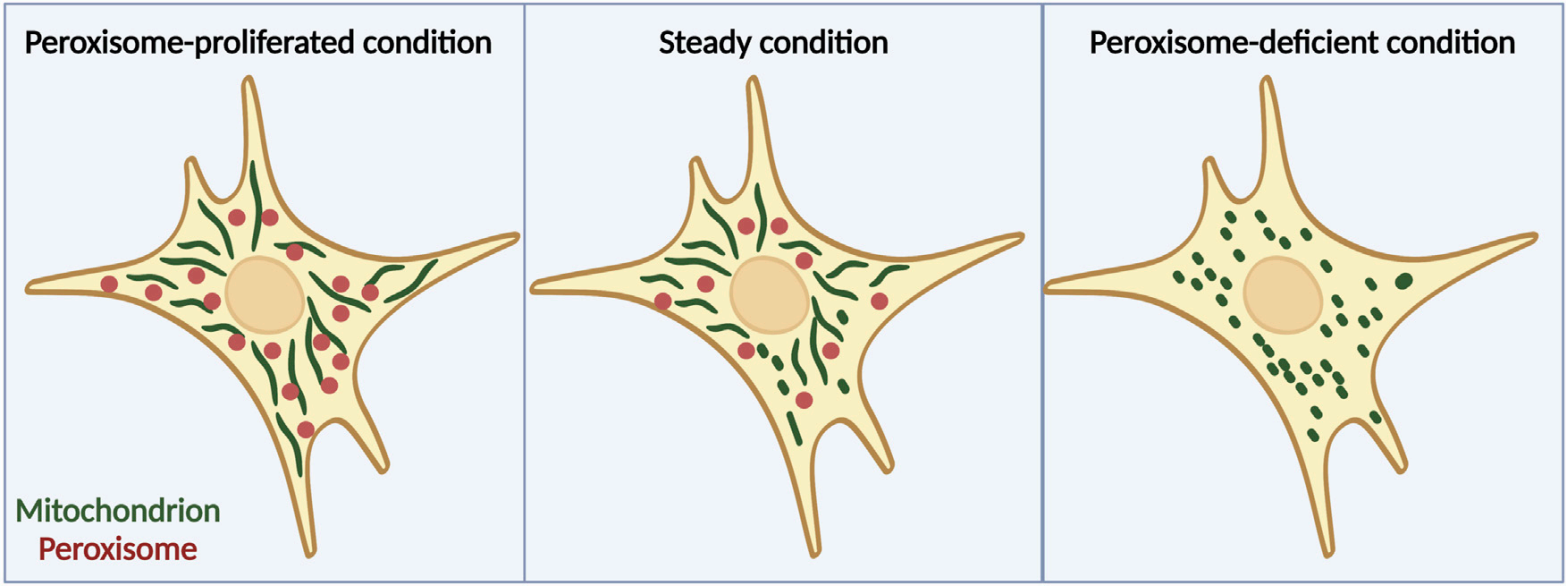
Peroxisomes are important for regulation of mitochondrial morphology. Mitochondrial fission-fusion dynamics in cells treated with 4-PBA (left), which induces peroxisome proliferation, cells under normal conditions (middle), and cells lacking peroxisomes due to Pex3 knockout (right). When peroxisome proliferation is induced pharmacologically, mitochondria acquire an elongated structure, whereas peroxisome deficiency leads to a more fragmented mitochondrial morphology. Note that in general, ZS only lacks peroxisomal matrix proteins but retains the membrane structures of peroxisomes. The figure is created with BioRender.com.

A recent study has suggested that peroxisomes are another factor that is involved in the regulation of mitochondrial dynamics (Tanaka et al., 2019). Peroxisomes have long been proposed to work together with mitochondria in many ways such as in β-oxidation of fatty acids to maintain lipid homeostasis and cellular ROS homeostasis (Schrader et al., 2015). Moreover, these two organelles reportedly share antiviral proteins and cooperate during viral infection (Dixit et al., 2010; Fransen et al., 2017). However, it has not been well investigated whether the interaction between peroxisomes and mitochondria affects their morphologies until recently. Our group reported that mitochondria become more fragmented under peroxisome-deficient conditions induced by acute depletion of Pex3 by using MEFs derived from Pex3fl/fl; Rosa-Cre-ERT2 mice, which are homozygous for a floxed allele of Pex3 and harbor a tamoxifen-inducible transgene for Cre recombinase or acute depletion of Pex5 by CRISPR-Cas9 system (Tanaka et al., 2019). By contrast, they become more elongated when the number of peroxisomes is increased pharmacologically by treatment with 4-PBA (which induces peroxisome proliferation) (Tanaka et al., 2019), implying that peroxisomes can control mitochondrial morphology (Figure 1). Besides, under peroxisome-depleted conditions, Drp1 localizes to mitochondria more than WT cells, and introducing catalytically inactive Drp1 (K38A mutant) (Frank et al., 2001) to Pex3 KO MEFs can rescue the fragmentation of mitochondria (Tanaka et al., 2019). These findings suggest that mitochondrial fragmentation induced by depletion of peroxisome is mediated by the translocation of Drp1 from peroxisomes to mitochondria.

Another study by Youle’s group also suggested that peroxisome-mitochondria interactions are involved in regulation of mitochondrial morphology. They revealed that depletion of VPS13D (vacuolar protein sorting 13D), which regulates mitochondrial fission and fusion, leads to an increase in round-shaped mitochondria and complete or partial loss of peroxisomes (Baldwin et al., 2021). However, it is still unclear how “loss of peroxisomes” and “abnormal mitochondrial morphology” are related under VPS13D KO condition. Given our observation that loss of peroxisomes promotes mitochondrial fission by promoting mitochondrial localization of Drp1, a causal relationship may be established, i.e., loss of peroxisomes caused by depletion of VPS13D increases mitochondrial fission through increased Drp1 localization. Future studies are required to study how Drp1 behaves under VPS13D depletion.

### Tethering mechanism between peroxisomes and mitochondria

Many researchers have studied the MCSs between peroxisomes and mitochondria. Like other organelle contacts, an outer membrane protein-mediated tethering mechanism underlies peroxisome-mitochondria interactions (Eisenberg-Bord et al., 2016). In line with this idea, several organelle tethering mechanisms function in peroxisome-mitochondria communication.

The precise tethering mechanism was first described in yeast, and peroxisomal Pex11 was anticipated to contact ERMES (ER-mitochondria encounter structure in yeast, a tethering complex of MERCs) protein Mdm34 (Mattiazzi Ušaj, 2015). A subsequent systematic study using the split-Venus system revealed that the yeast mitofusin Fzo1 and PMP Pex34 may function as tethers at the peroxisome side (the binding partners of Fzo1 or Pex34 have not been explored) (Shai et al., 2018). In this study, Shai et al. observed that deletion of Fzo1 did not affect the tethering function of Pex34 and vice versa. Therefore, it was suggested that multiple pairs of tethering complexes function in communication between peroxisomes and mitochondria (Shai et al., 2018).

Papadopoulos et al. also showed that peroxisome-mitochondria interactions are promoted during hormone biosynthesis in mammalian cells (Fan et al., 2016). They reported that induction of steroidogenesis by dibutyryl cAMP in Leydig cells rapidly triggers peroxisomes to approach mitochondria and contact is mediated by isoform A of the acyl-CoA-binding protein ACBD2/ECI2 (Fan et al., 2016). Moreover, they revealed that ACBD2/ECI2 contains mitochondria- and peroxisome-targeting sequences at its N-terminus and C-terminus, respectively (Fan et al., 2016), suggesting that dual targeting of ACBD2/ECI2 may help to establish two-way communication between these two organelles (Islinger et al., 2018). It would be interesting to examine whether ACBD2/ECI2 acts as a tethering protein in other biological contexts.

Though several tethering mechanisms between peroxisomes and mitochondria are reported in yeast and mammalian cells, whether they are involved in regulating mitochondrial morphology has been elucidated. Given that the tethering mechanism has been suggested to regulate mitochondrial fission in lysosomes-mitochondria interplay (Wong et al., 2018), it is tempting to hypothesize that tethering molecules between peroxisomes and mitochondria also control mitochondrial dynamics. Testing this hypothesis would be of interest in the future studies.

### Association of disrupted mitochondrial fission-fusion regulation with Zellweger syndrome

Patients with ZS possess severe defects in many organs, such as the brain, muscle, liver, and kidney. In addition, they exhibit various clinical features including hypotonia, craniofacial dysmorphia, skeletal weakness, growth retardation, intellectual disability, spasticity, seizures, and vision and hearing failure (Trompier et al., 2014). Notably, dysfunctions of mitochondrial fission-fusion have also been linked to neuronal abnormalities, muscle atrophy, and impaired osteogenesis (Detmer and Chan, 2007; Chen et al., 2010; Romanello et al., 2010; Touvier et al., 2015; Forni et al., 2016). For instance, Mfn2-knockout (KO) Purkinje cells exhibit aberrant mitochondrial ultrastructures and cellular degeneration (Chen et al., 2007), which are some of the most prominent features of ZS (Barry and O’Keeffe, 2013; Trompier et al., 2014). In addition, muscle-specific Drp1 overexpression impairs skeletal growth during myogenesis (Touvier et al., 2015), overexpression of Drp1 or Fis1 in the *tibialis anterior* is sufficient to activate muscle wasting (Romanello et al., 2010), and conditional deletion of Mfn1 or Mfn2 causes atrophy in differentiated skeletal muscle (Chen et al., 2010). Moreover, knockdown of Mfn2 in murine mesenchymal stem cells during osteogenesis results in loss of the ability of these cells to differentiate into osteocytes (Forni et al., 2016).

Although the defects observed in ZS and those observed in mice with disrupted mitochondrial dynamics have similarities, the cellular basis for the pathogenesis of ZS remains unclear. Given that the absence of peroxisomes can lead to a fragmented mitochondrial morphology both in flies (Bülow, 2018) and mammalian cells (Tanaka et al., 2019), the pathology of ZS may be explained by impaired mitochondrial fusion or excess mitochondrial fragmentation caused by a dysfunction or lack of peroxisomes.

## Peroxisome-dependent regulation of apoptotic pathways

Apoptosis is a programmed cell death that contributes to cellular maintenance, development, and defense against cellular stresses including infection (Sheridan and Martin, 2010). There are two apoptotic pathways: an extrinsic pathway and an intrinsic pathway. In the intrinsic apoptotic pathway, mitochondria play an important role by releasing pro-apoptotic factors such as cytochrome c from their intermembrane spaces through a pore composed of the Bcl-2 family proteins BAX and BAK (Sheridan and Martin, 2010; Tait and Green, 2010). Recent studies suggest that peroxisomes are involved in the mitochondria-dependent apoptotic pathways. This section will summarize the recent findings on the possible roles of peroxisomes in regulating intrinsic apoptotic pathways.

### Peroxisomes affect apoptotic pathways through redox control

Peroxisomes are known to function as an essential intracellular signaling platform in redox homeostasis (Schrader et al., 2015). Furthermore, several studies also indicate that redox control by peroxisomes may affect mitochondria-dependent cell death. Wang et al. (2013) showed that excessive peroxisomal ROS production elicits mitochondria-mediated cell death possibly through redox communication between peroxisomes and mitochondria, and that the presence of functional peroxisomes guards cells against this oxidative stress-induced apoptosis (Wang et al., 2013). Their findings may imply that the controlled redox homeostasis by peroxisomes protects cells from intrinsic apoptotic pathways. Another study by Hosoi et al. revealed that apoptotic protein BAK can localize not only to mitochondria but also to peroxisomes, and the peroxisomal BAK releases catalase from peroxisomes into the cytosol (Hosoi et al., 2017). The released catalase may contribute to redox homeostasis by eliminating H_2_O_2,_ thus protecting cells from cell death such as apoptosis.

### Peroxisomal deficiency promotes caspase activation and apoptosis

Abnormal mitochondrial morphology, such as mitochondrial fragmentation and collapsed cristae, is associated with leakage of cytochrome c (Suen et al., 2008; Otera et al., 2016). Leaked cytochrome c forms a complex called the apoptosome with Apaf1 (apoptotic protease-activating factor 1) and the initiator caspase caspase-9 in the cytoplasm (Hyman and Yuan, 2012). Caspase-9 cleaves and activates executioner caspases such as caspase-3 and caspase-7, leading to mitochondria-dependent apoptosis (Hyman and Yuan, 2012). The division process of mitochondria mediated by Drp1 has been suggested to play an essential role in promoting mitochondrial apoptotic pathways (Westermann, 2010). As described earlier, peroxisomes regulate mitochondrial dynamics and a deficiency of peroxisomes can cause abnormal mitochondrial morphology.

**FIGURE 2:**
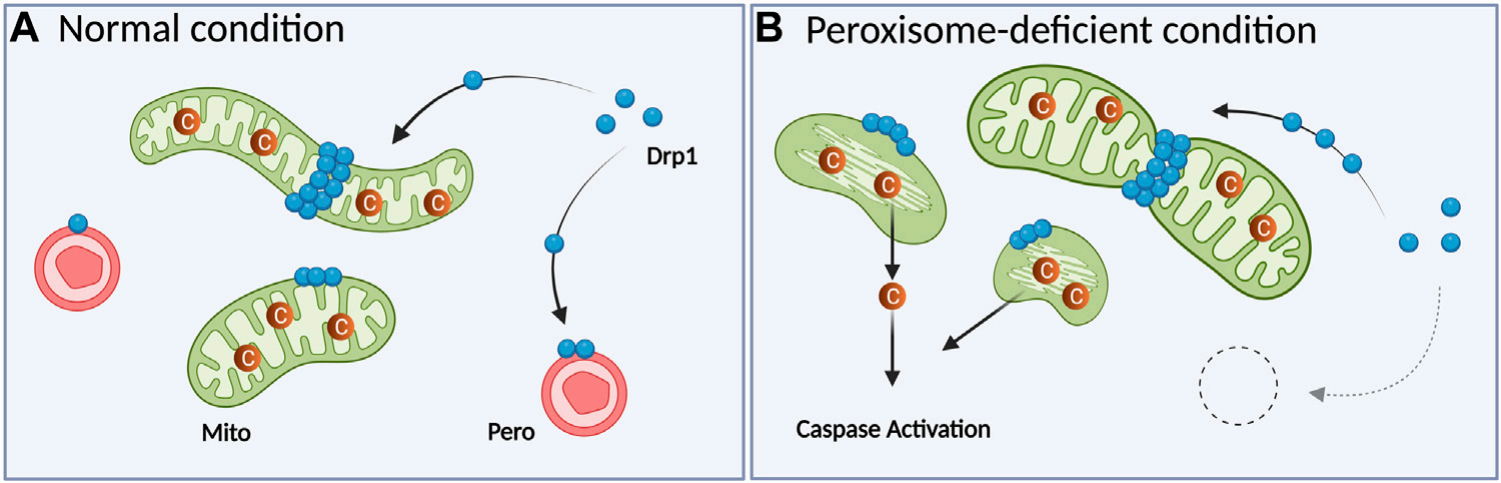
The absence of peroxisomes results in excess mitochondrial fragmentation following caspase activation in mammalian cells. **(A)** In normal conditions, cytosolic Drp1 is recruited to both peroxisomes and mitochondria. **(B)** When peroxisomes are acutely depleted by Pex3 KO, Drp1 is recruited to mitochondria more than WT cells. Translocated Drp1 will induce mitochondrial fragmentation along with abnormal cristae structures. This fragmentation induces the release of cytochrome c to the cytosol, resulting in caspase activation. Mito: mitochondrion, Pero: peroxisome, C: cytochrome c. The figure is created with BioRender.com.

Association of peroxisomal deficiency with cell death has been illustrated in several ways. In PBD patients and model mice in which peroxisomes are dysfunctional or absent (Trompier et al., 2014), increased apoptosis is also observed. Cultured cerebellar neurons from Pex13-null mice (a ZS model) display enhanced oxidative stress and apoptosis together with mitochondrial dysfunctions (Müller, 2011). A similar observation was made in heterozygous and homozygous Pex11β-KO mice; primary neuron cultures from the cortex and cerebellum of these mice exhibit increased cell death (Ahlemeyer et al., 2012). Besides, Pex5 KO or cKO exhibited increased apoptosis in the neocortex or cerebellum, respectively (Baes et al., 1997; Krysko et al., 2007). However, these studies were conducted using long-term depletion of peroxisomes. Since elevated ROS is connected with mitochondrial fragmentation (Rakovic et al., 2011; Wu et al., 2011) and apoptosis (Simon et al., 2000), accumulated ROS could possibly induce apoptosis as a secondary effect. Thus, it was unclear whether the phenotypes of human patients and model animals reflect the primary effect of peroxisome deficiency or secondary effects. Recently, peroxisome deficiency caused by acute Pex3 KO was revealed to increase the level of cytosolic cytochrome c and enhance caspase activity without inducing apoptosis, whereas DNA damage in Pex3-KO cells elevated caspase activation and increased the number of cells undergoing apoptosis (Tanaka et al., 2019). In this study, localization of the division machinery Drp1 to mitochondria was increased under peroxisome deficiency and the introduction of catalytically inactive Drp1 (K38A mutant) rescued the cytochrome c diffusion (Tanaka et al., 2019). These results are consistent with the previous findings that Drp1 recruitment to mitochondria is important not only for increasing mitochondrial fission, but also for releasing cytochrome c (Frank et al., 2001; Breckenridge et al., 2003; Lee et al., 2004; Germain et al., 2005; Youle and Karbowski, 2005). These observations suggest that Drp1 can mediate mitochondrial fragmentation and subsequent cytochrome c release when peroxisomes are lacking (Figures 2A,B).

## Concluding remarks and future perspectives

We summarized that the functions of peroxisome-mitochondria interactions are not limited to well-known fatty acid catabolism, but also regulate mitochondrial fission-fusion dynamics and mitochondrial apoptotic pathways. Patients with PBDs exhibit abnormal mitochondrial morphologies and enhanced apoptosis in several cell types. Therefore, elucidation of the novel functional roles of peroxisomes in mitochondrial fission-fusion dynamics and apoptotic pathways may improve understanding of the pathogenesis of PBDs. Notably, stresses such as ultraviolet irradiation and an elevation of ROS are known to increase the volume of intracellular peroxisomes (van der Valk et al., 1985; Schrader and Fahimi, 2004; Schrader and Fahimi, 2006). We assume that cells counteract stresses by increasing the number of peroxisomes, thereby inhibiting mitochondrial fragmentation and subsequent caspase activation. In addition, fatty acids such as oleic acid and high-fat feeding increase the number of intracellular peroxisomes; therefore, cellular fatty acid metabolism may alter stress sensitivity (Ishii et al., 1980; Diano et al., 2011). The relationship between cell types in which fatty acid synthesis is high (e.g., adult neural stem cells) and stress tolerance is also an exciting issue to be tackled (Knobloch et al., 2013).

We proposed that mitochondrial fragmentation and subsequent cytochrome *c* releasement were in part mediated by elevated recruitment of Drp1 to mitochondria in peroxisome-deficient cells. However, the molecular mechanism by which mitochondrial fragmentation induced by Drp1 triggers the releasement of cytochrome *c* in peroxisome-deficient cells remains unknown. Besides, the relationship between the Drp1-mediated pathway and the possible tethering molecules remains elucidative. Future studies examining these points will provide new insights into the involvement of peroxisomes in mitochondrial morphology and functions.
